# P-1690. The relationship of prescriber role and timing of prescribing to antibiotic prescribing appropriateness at a tertiary academic hospital

**DOI:** 10.1093/ofid/ofae631.1856

**Published:** 2025-01-29

**Authors:** Paul Greidanus, Ryan LeBlanc, Dima Kabbani, Stephanie W Smith, Karen Doucette, Cecilia Lau, Serena Bains, Karen G Fong, Jackson J Stewart, Teagan Zeggil, Justin Chen

**Affiliations:** University of Alberta, Edmonton, Alberta, Canada; University of British Columbia, Victoria, British Columbia, Canada; University of Alberta, Edmonton, Alberta, Canada; University of Alberta, Edmonton, Alberta, Canada; University of Alberta, Edmonton, Alberta, Canada; Alberta Health Services, Edmonton, Alberta, Canada; Alberta Health Services, Edmonton, Alberta, Canada; Alberta Health Services, Edmonton, Alberta, Canada; Alberta Health Services, Edmonton, Alberta, Canada; Alberta Health Services, Edmonton, Alberta, Canada; University of Alberta, Edmonton, Alberta, Canada

## Abstract

**Background:**

There are limited studies examining antibiotic prescription appropriateness that compare trainees versus attending physicians or time of prescription in relation to working hours. Antimicrobial stewardship (AMS) prospective audit and feedback (PAF) was implemented in 2018 at a tertiary care centre targeting six restricted antibiotics (carbapenems, daptomycin, linezolid, and tigecycline). The agent, regimen, and duration were assessed by AMS pharmacists and/or physicians against institutional prescribing guidelines (expert opinion if not available) and recorded prospectively in the AMS quality improvement database. Real time written and verbal feedback were provided to the most responsible physician.

Table 1

**Figure:**
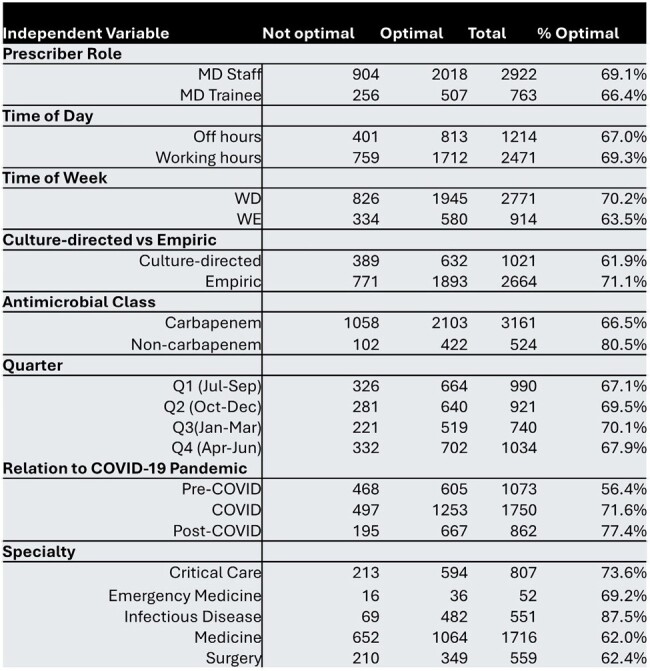

Baseline prescription demographics.

**Methods:**

We examined all prescriptions subjected to PAF from 2018 to 2023, with the primary objective to assess prescription appropriateness for medical trainees versus staff physicians. Secondary objectives were to evaluate whether other variables including time of day (working vs off hours), weekday vs weekend, and the COVID-19 pandemic period (Mar 2020-May 2022) impacted appropriateness. Multiple logistic regression was used to determine factors independently associated with optimal prescribing of antibiotics. Institutional ethics approval was obtained.

Figure 1

**Figure:**
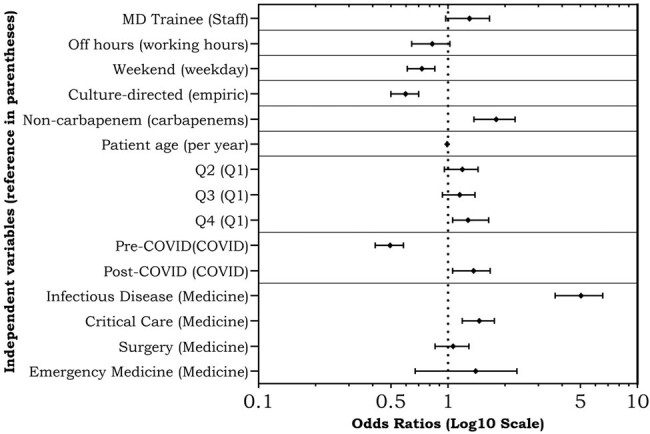

Odds ratios of prescription appropriateness for each independent variable (reference in parentheses).

**Results:**

Overall, 3685 prescription audits were included in this study. Baseline prescription demographics are shown in Table 1. Of these, 1106 (32%) audited prescriptions were assessed by AMS PAF as not optimally prescribed requiring actionable AMS intervention. Prescriptions written by trainees were not less appropriate compared to staff (OR 1.27 [95%CI 0.97-1.66]). Off hours prescriptions had lower odds of being optimal (OR 0.81 [0.64-1.02], NS). Other secondary outcomes are reported in Figure 1. Prescriptions initiated by infectious disease (ID) had higher odds of being optimal (4.88 [3.68-6.55]).

**Conclusion:**

In our cohort, we did not find a difference in appropriateness of restricted antibiotic prescriptions between trainees and staff, with a trend towards decline in optimal prescribing during off hours and weekends. ID had higher odds of optimally prescribing. Appropriateness improved over time and the COVID-19 pandemic did not have a negative effect at our center.

**Disclosures:**

**All Authors**: No reported disclosures

